# Deformation Behavior of Elastomer-Glass Fiber-Reinforced Plastics in Dependence of Pneumatic Actuation

**DOI:** 10.3390/biomimetics6030043

**Published:** 2021-06-22

**Authors:** Mona Mühlich, Edith A. González, Larissa Born, Axel Körner, Lena Schwill, Götz T. Gresser, Jan Knippers

**Affiliations:** 1 Institute of Building Structures and Structural Design (ITKE), University of Stuttgart, Keplerstrasse 11, 70174 Stuttgart, Germany; edith-anahi.gonzalez-san-martin@itke.uni-stuttgart.de (E.A.G.); axel.koerner@itke.uni-stuttgart.de (A.K.); jan.knippers@itke.uni-stuttgart.de (J.K.); 2 Institute for Textile and Fiber Technologies (ITFT), University of Stuttgart, Pfaffenwaldring 9, 70569 Stuttgart, Germany; larissa.born@itft.uni-stuttgart.de (L.B.); lena.schwill@itft.uni-stuttgart.de (L.S.); goetz.gresser@itft.uni-stuttgart.de (G.T.G.); 3 German Institutes of Textile and Fiber Research (DITF), Koerschtalstrasse 26, 73770 Denkendorf, Germany

**Keywords:** fiber-reinforced plastic (FRP), elastomer, pneumatics, actuation, folding, finite element modeling (FEM), simulation

## Abstract

This paper aims to define the influencing design criteria for compliant folding mechanisms with pneumatically actuated hinges consisting of fiber-reinforced plastic (FRP). Through simulation and physical testing, the influence of stiffness, hinge width as well as variation of the stiffness, in the flaps without changing the stiffness in the hinge zone, was evaluated. Within a finite element model software, a workflow was developed for simulations, in order to infer mathematical models for the prediction of mechanical properties and the deformation behavior as a function of the aforementioned parameters. In conclusion, the bending angle increases with decreasing material stiffness and with increasing hinge width, while it is not affected by the flap stiffness itself. The defined workflow builds a basis for the development of a predictive model for the deformation behavior of FRPs.

## 1. Introduction

According to the European Commission, buildings are accountable for around 40% of the energy consumption in the European Union. Strategies to improve the energy performance of the built environment include the integration of building technologies that aim to regulate indoor comfort [1].

Façades are the interface between the interior and exterior of a building. Therefore, by responding to external climates and regulating the internal conditions, they become an essential element in creating energy-neutral buildings [2]. Since external environmental conditions and internal occupancy patterns are in constant change, the façade’s capability of adaptation plays a crucial role in improving the energy and environmental performance of a building.

Implementation of adaptive façades in Europe could lead to a 10% reduction of oil source energy and save 111 million tons of carbon dioxide per year [3]. On this account, the European Union’s vision of nearly zero-energy buildings recognizes the potential of adaptive façades as a positive influence on a building’s energy consumption [2,4].

Common implementations of adaptive façades rely on rigid body mechanisms to achieve the desired motion. However, in past years, less complex and promising solutions have derived from biomimetic principles. Prime examples are the current developments of adaptive façade elements that employ bio-inspired compliant mechanisms to drive kinematic behavior in the form of elastic deformation.

### 1.1. Previous Work

An early and well-known project based on this concept is flectofin^®^, inspired by the bird-of-paradise flower (*Strelitzia reginae*). Two lamellas consisting of fiber-reinforced plastic (FRP) are fixed to a stiff backbone and can bend up to 90° by applying force to the backbone. Novel at its time, this proved to be relatively limited in its kinematic behavior and fatigue strength due to the proportion of the modules to the actuators and the use of material with almost homogeneous stiffness that leads to high-stress concentrations in the FRP and high actuation forces [5].

Subsequent projects tried to overcome the limited fatigue strength by focusing on the development of folding elements with distinct flexible hinge zones. Flectofold was the first demonstrator to attempt this. For this project, the motion principle of *Aldrovanda vesiculosa* was abstracted into a curved-line folding mechanism for motion amplification with discrete curved hinge zones. The inflation of a pneumatic cushion located on the back of the midrib leads to bending of the midrib, and thereby to the amplified folding motion of the adjacent flaps which were connected to the midrib via hinge zones with reduced stiffness [6].

Current projects, such as the façade shading demonstrator Flexafold, have been inspired by the hindwings of insects. The wing-folding pattern of *Graphosoma lineatum italicum* was identified as flexagon principle and served as a basis for the geometry and folding pattern of Flexafold. Studies on the material set-up of hollow wing veins of the beetle *Dorcus titanus platymelus* were transferred into a FRP with a stiffness gradient from a flexible hinge zone to stiffer areas subjected to angular displacement, enabling the technical implementation of the Flexafold demonstrator [7,8].

The most recent example of the research work in this field is the ITECH Research Demonstrator 2018–19, which showcased the possibility of industrial manufacturing of large-scale folding elements. The motion is actuated by pneumatic cushions integrated into distinct flexible hinge zones. Taking as inspiration the folding behavior of ladybug wings (*Coleptera coccinellidae*), the zones next to the hinge areas increase in thickness, creating a reinforcement with higher stiffness in the critical zones [9].

### 1.2. Biomimetic Background

The research work presented is linked directly to the described research projects and demonstrators. Both the materiality and the actuation principle have been abstracted from the motion and actuation behavior of insect wings: in many species, the folding motion is realized by fibrous material which is actuated by internal pressure change.

**Figure 1:**
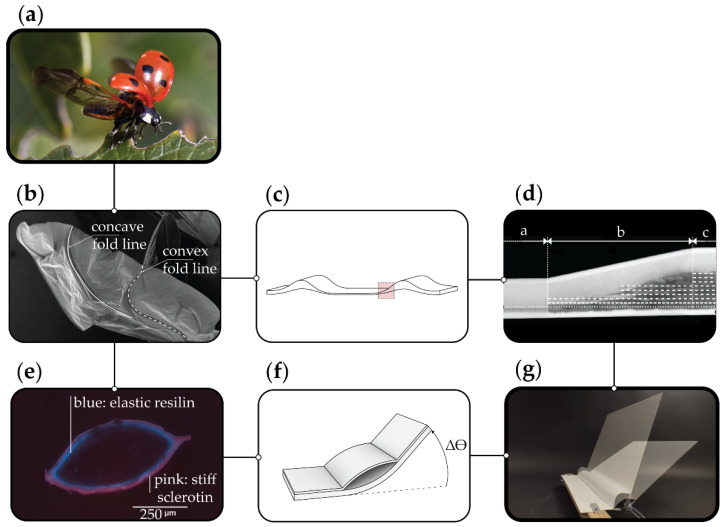
Biomimetic abstraction process with (**a**) biological role model *Coleoptera coccinellidae* [10], (**b**) scanning electron microscope image of the hindwing [11], (**c**) schematic of the material cross section in the insect wing (adapted from [7]), (**d**) hybrid FRP with graded material transitions between stiff and flexible zones [12], (**e**) autofluorescence image of the hollow vein in insect wing with marked sclerotin (pink) and resilin (blue) (adapted from [7]), (**f**) abstracted actuation principle with stiffer (bottom) and less stiff laminate and (**g**) FRP combining both principles (material and actuation).

The abstraction process, which underlies the present work, is shown in Figure 1. In particular, the ladybug wing (Figure 1a) was investigated as biological role model. The fragile hind wings of the beetle are shown in the scanning electron microscope (SEM) image (Figure 1b).

Two principles of the insect wing were abstracted and transferred into a demonstrator (Figure 1g). First principle is the material set-up itself based on the pliable zones of the wing: Next to the local, compliant folding zones, respectively the hinge zones of the wing, there are reinforced, stiffer zones. The thickening is formed on one side of the wing. This determines the folding direction of this pliable zone in the wing. Figure 1c shows a simplified representation. Instead of using different materials in the biological role model, just one material is used for the stiff and flexible zones. This material concept was abstracted and transferred into a FRP (Figure 1d). The FRP consists of elastomer and glass fiber-reinforced plastic (GFRP), covered and sealed with a thermoplastic polyurethane (TPU) film. These three materials form one hybrid FRP in which the stiffness can be adjusted by the cross-section-related content of GFRP.

**Figure 2:**
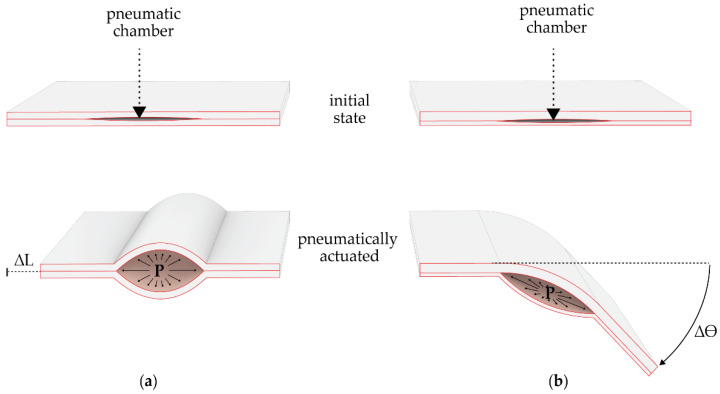
Actuation principle of elements with embedded pneumatic actuators with (**a**) plates with identical stiffness, leading to a shortening in length and (**b**) elements with plates of asymmetrical stiffness causing an angular displacement (adapted from [8]).

The second principle is the folding mechanism based on the hollow wing veins (Figure 1e) [8]. The hollow wing is enclosed by resilin (flexible) and sclerotin (stiff) in different proportions on the upper and lower side of the hollow vein. This determines the bending direction when the vein is pressurized (Figure 1f and Figure 2). Both principles were unified in a demonstrator shown in Figure 1g.

The demonstrator is the starting point for the investigations presented within the paper: the physical testing and simulation of FRP elements with asymmetric laminate set-ups. The embedded pneumatic actuation of the hinge will lead to an asymmetric elastic deformation, causing a rotation towards the less stiff side of the element (Figure 2). The aim of further exploring this kinematic principle is to create adaptive façade elements capable of achieving a high range of motions, yet robust enough to withstand the external forces acting on a building’s skin.

## 2. Materials and Methods

### 2.1. Elastomer-Glass Fiber-Reinforced Plastic (E-GFRP) Hybrid Laminate

Based on preliminary research [8,13,14] regarding actuated FRPs, a combination of elastomer, GFRP and TPU film is used. The stiffness ratio between the laminate above and below the actuation chamber is adjusted by the GFRP content in the laminate set-up. Two intermediate elastomeric layers surrounding the cushion ensure the interlaminar adhesion between the upper and lower laminate once the actuation chamber expands. For protection against external environmental influences (UV radiation, water absorption, etc.), the laminate is coated with a TPU film. The material properties of the different layers are listed in Table 1.

**Figure 3:**
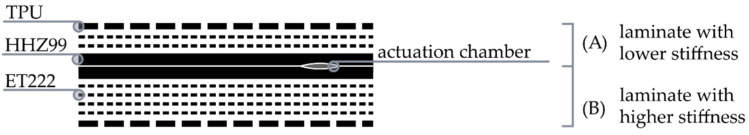
Schematic of an exemplary laminate set-up (TPU/ET222_4_/HHZ99_2_/ET222_2_/TPU), where ET222 is implemented with a fiber orientation of ±45°.

Figure 3 shows an example of a laminate set-up of such an actuatable FRP. In the figure, the stiffness of the laminate above the actuation chamber is lower. The expansion of the chamber would cause the entire laminate to bend accordingly in the direction of this laminate.

### 2.2. Simulation of E-GFRP Hybrid Laminates

The simulations are based on the third-order theory with a stiffness update after each iteration. For the simulation workflow a three-dimensional model of the system is developed as input geometry for the nonlinear FEM simulation (SOFiSTiK Version 2018, SOFiSTiK AG, Oberschleißheim, Germany). The parametric model is discretized into quad shell elements and the geometry converted into elements with the needed information about, e.g., beams, couplings and supports for SOFiSTiK.

Also, the physical material characteristics set the boundaries for the simulations. First, the material behavior was calculated with the program Helius Composite 2017 by Autodesk. The composite type, the manufacturing properties and the fiber direction of each layer were set in order to calculate the characteristics of the compound materials and to define the thickness of the material in each area. Secondly, the interlaminar adhesion strength of the materials predetermined the maximal pressure for the actuation. The data basis for the materials was taken from Born’s previous work [12].

**Figure 4:**
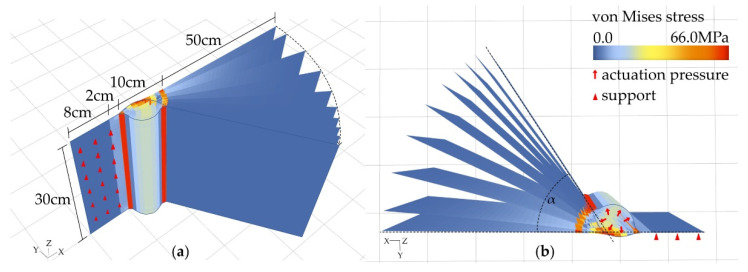
Simulation principle shown on test with 2 + 4 layers and a hinge width of 10 cm, (**a**) perspective view and (**b**) top view.

To compare the simulation to physical tests, a consistent geometry of 50 cm × 30 cm was chosen for both physical and digital simulation. The geometry was then modeled in the FEM software SOFiSTiK. The element was fixed in all degrees of freedom on one side, to set it up as in the physical tests where it was clamped likewise. The fixation had an offset of 2 cm to the start of the hinge (Figure 4a,b).

The tests investigated the influence of three different parameters on the resulting bending angle. The maximal actuation was defined by the maximal adhesion strength of the core material around the hinge, which for all tests was the elastomer HHZ99 with F_max = 477 N/10 cm [12].

### 2.3. Pneumatic Actuators

**Figure 5:**
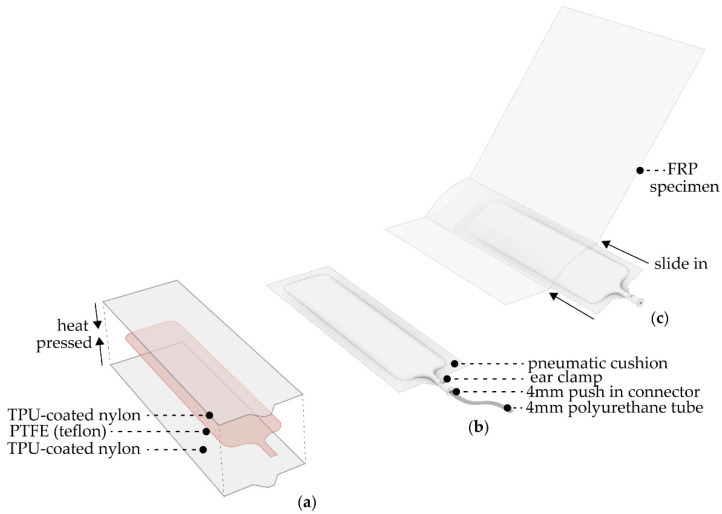
Configuration of pneumatic actuator exemplifying (**a**) materiality, (**b**) pneumatic fixtures and (**c**) integration of pneumatic cushion to composite specimen.

The motion is actuated by pneumatic cushions integrated within the hinge zone. The pneumatic actuators are fabricated with a 70 den (170 g/m^2^) coated Nylon. This is a lightweight and airtight fabric that is coated on one side with a thermoplastic polyurethane that allows it to be heat sealable. The pneumatic actuators’ fabrication starts by creating the desired shape with a PTFE film; this is later placed between two layers of TPU-coated nylon and heat pressed (Figure 5a).

This process will bond the edges surrounding the PTFE, resulting in a pneumatic cushion that can be inflated and deflated as needed. The shape of the cushion follows the dimension of the hinges created on the FRP specimens. However, in all instances the cushions are made 2 cm wider than the hinge, to ensure that they will fill in the volume created during actuation. The corners of the cushions are rounded, and a thin linear piece extends to create the cushion’s inlet. For the air inlet, a pneumatic push-in connection is secured with an ear clamp to make it airtight. As previously mentioned, the composite elements are pressed with a piece of Teflon film in between layers. This creates a chamber in the component that allows for the insertion of the cushion. The cushion is inserted by hand, making sure it is evenly distributed through the chamber (Figure 5).

**Figure 6:**
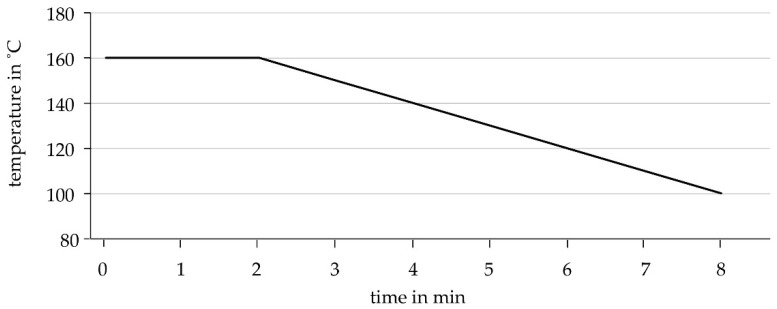
Temperature curve in pressing cycle at constant pressure of 1.3 bar.

For this research, the cushions were pressed with two different techniques: with an industrial heat-press and manually with a regular flat iron. It was observed that the cushions made with the industrial heat-press proved to be less prone to leakage and more durable due to the absence of human error and the systematic production with defined pressure ranges, temperature, and time of pressing (Figure 6).

### 2.4. Actuation and Bending Test Set-Up

**Figure 7:**
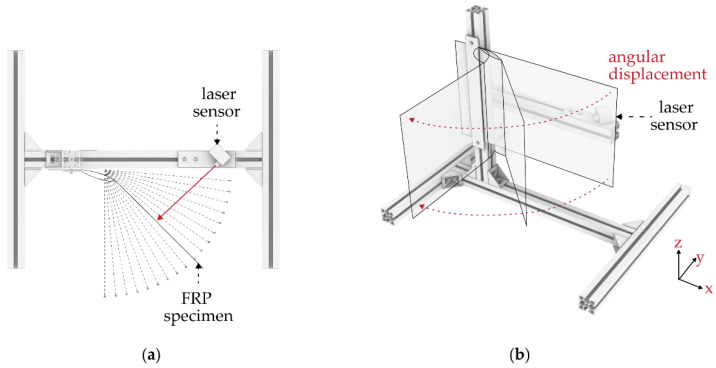
Vertical test set-up, with specimen clamped on one side to allow for the angular displacement (**a**) top view and (**b**) axonometric.

The specimens were tested in a set-up that would allow the angular displacement of the element. The test set-up was designed to assess the specimens vertically to prevent gravitational forces from interfering in the results (Figure 7). The aim of these tests was to extract the air pressure needed in the pneumatic actuator for different bending angles and subsequently analyze the performance of the material.

**Figure 8:**
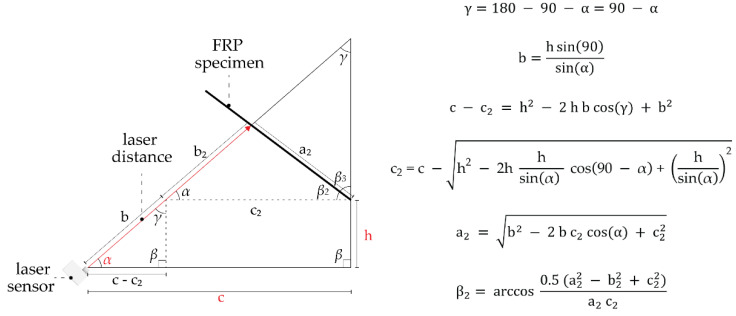
Trigonometric functions to find the angular displacement, when h, c and α are the input variables (adapted from [15]).

The test set-up consists of a clamping area in which the specimen is fixed at one edge. When the integrated pneumatic cushion is actuated, the asymmetric material layup will enable the bending of the hinge, causing an angular displacement towards the direction of the thinner layer. A distance laser sensor placed on the opposite side of the clamping area will give the distance to the element. With this measurement, the element’s final bending angle can be calculated by using a set of trigonometric functions. The measurement errors have not been considered, because they are mostly systematic and for the comparison of the results within our set up thereby of less impact (Figure 8) [15].

The system works by setting up the desired bending angle; this enables a compressor that inflates the pneumatic cushion until the desired angle is achieved. After running the tests for the required number of cycles, one can extract the reading of the bending angles and the exact air pressure needed to reach them.

## 3. Results

### 3.1. Simulative Investigation of the Geometrical Parameters of an Actuated Hinge

For the following results, we changed the stiffness, the hinge width and the material in the surface area, compared to the hinge, taking minimal three variations into consideration. An overview of the tests and their compositions can be seen in Table 2.

**Figure 9:**
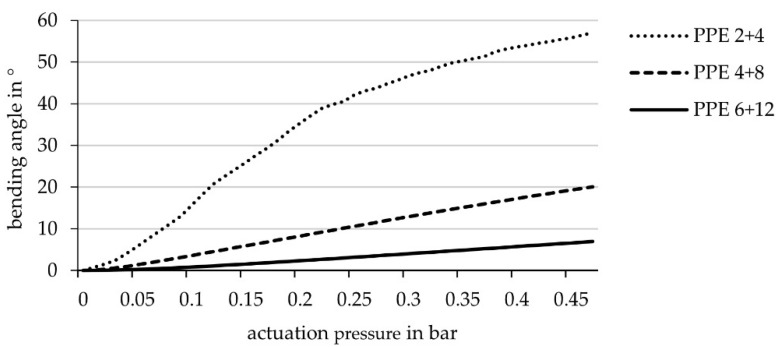
Achievable bending angle (simulation) in relation to the actuation pressure for laminate set-ups with different stiffness’s: 2 + 4 layers ET222 (laminate set-up of PPE 2 + 4 similar to Figure 2), 4 + 8 layers ET222 and 6 + 12 layers ET222, hinge width 10 cm.

The first simulations compare the bending angles of three different total stiffnesses: two layers of ET222 on one side of the hinge and four on the other side and, respectively, four and eight layers as well as six and twelve layers. The thinnest lamina resulted in the largest bending angle of the element, and therefore was chosen for the subsequent tests. The three maximal bending angles suggest a negative exponential correlation between material thickness and bending angle (Figure 9).

**Figure 10:**
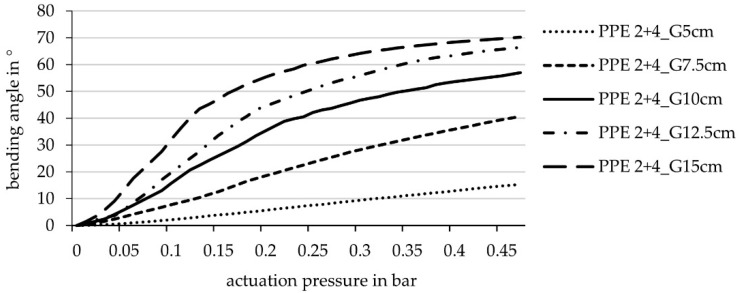
Achievable bending angle (simulation) in relation to the actuation pressure for laminate set-ups with different hinge widths: 5 cm, 7.5 cm, 10 cm, 12.5 cm, 15 cm (laminate set-up PPE 2 + 4 cf. Figure 1 equal for all samples).

The second series of tests investigate the influence of hinge width. The investigated hinge widths are based on experience values from previous research work and experiments [8,9]. The hinge width was increased by 2.5 cm steps, ranging from 5 cm to 15 cm width. The results showed a big divergence of in the maximal bending angle between the 5 cm (15.3°), the 7.5 cm (40.7°) and the 10 cm wide hinge (57°), while the difference from the 12.5 cm width (66.4°) to the 15 cm wide hinge (70.2°) was comparatively small (Figure 10).

**Figure 11:**
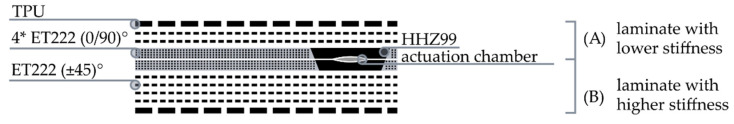
Schematic sketch of laminate set-up with stiffened flaps due to the substitution of HHZ99 by four layers ET222 (fiber orientation 0/90°) (HHZ99 layer on laminate side (**A**) with lower stiffness 5 cm wider than on laminate side (**B**) with higher stiffness).

In the third test, the elastomer in the flaps is replaced by four layers of ET222, which corresponds to the thickness of one layer of HHZ99. The orientation of these ET222 layers is shifted by 45° (compared to the continuous ET222 layers), resulting in an effective fiber direction of (0/90)° in relation to the bending axis (Figure 11).

**Figure 12:**
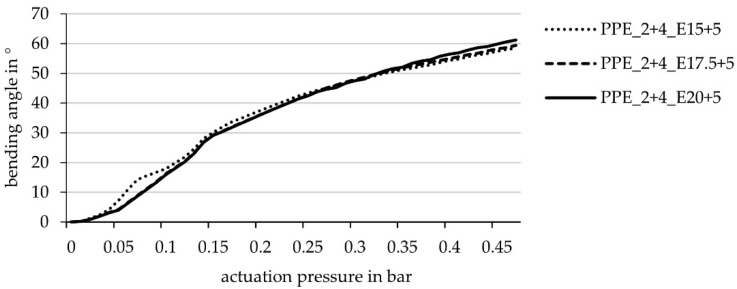
Achievable bending angle (simulation) in relation to the actuation pressure for laminate set-ups with different widths of the elastomeric layers enclosing the cushion chamber (width 10 cm): 15 + 5 cm, 17.5 + 5 cm, 20 + 5 cm (elastomer layer on the side with lower stiffness 5 cm wider than on the side with higher stiffness, cf. Figure 11).

In the simulation, variations of the elastomer width around the hinge were calculated with the set-up of the thin lamina and 10 cm hinge width. The difference of the bending angle proved to be very small. However, compared to the previous results for the thin lamina with a 10 cm hinge width, the actuation curve starts steeper and ends at a slightly higher bending angle (Figure 12).

### 3.2. Mechanical Investigation of the Geometrical Parameters of an Actuated Hinge

**Figure 13:**
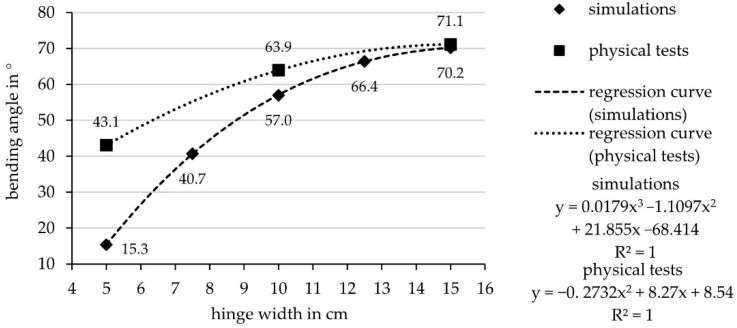
Regression formula with a polynomic approximation curve based on the results of the simulations and the physical tests.

In addition to the simulative, theoretical examination, the abovementioned laminate set-ups were also tested mechanically by means of test components. The size of the flap of the specimens to be actuated was 30 × 30 cm. It can be noted that the maximum deformation angle of the specimens almost matches the simulation results (Figure 13).

However, the deformation angle resulting from the simulation is generally smaller than that of the real mechanical test, because the physical tests have been actuated till the failure of the material, which emerged to be a higher pressure than in the simulations. This is caused by the data regarding the interlaminar adhesion on which the simulation and the failure criteria are based. The data on interlaminar adhesion result from T-peel tests in which the plies are peeled off from each other at a 90° angle. However, when the vacuum chamber is actuated, a much smaller angle is created between the plies, visible in the larger discrepancies of the smaller hinges where higher pressures than expected, could be applied. Thus, the limit chosen for the simulations is relatively low and probably can be set higher. Thereby, also the actuation pressure and bending angle would be higher in the simulations and the results of simulation and practical testing would converge.

### 3.3. Component Actuated with Multiple Cushions

Based on the investigations presented in Section 3.1, it can be stated that a bending angle of 90° cannot be achieved with only one cushion. A larger bending angle could generally be achievable by combining several cushions next to each other. The regression analysis of the data of the previous test specimens (variation of the hinge width) should predict the necessary hinge width for a deformation of 90°.

#### 3.3.1. Regression Model from Simulative Investigation

For the regression formula (Figure 13), a polynomic approximation curve was used as it fitted the results best; with the formula predictions about the resulting bending angles for varies hinge widths were made (Table 3).

**Figure 14:**
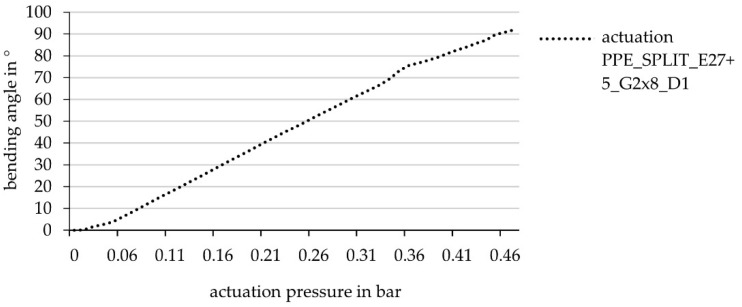
Results of simulative testing of an element with two hinges (hinge width 8 cm, distance between hinges 1 cm), bending angle in relation to actuation pressure.

The interpolation for the angle of 45° yielded a hinge width of 8.14 cm. Hence, for the simulation of the deformation behavior, two hinges with a hinge width of 8 cm were used to reach a bending angle of approximately 90°.The results of the simulation regarding the deformation behavior of a sample with two cushions and a hinge width of 8 cm are shown in Figure 14.

It can be said that the bending angle of 90° is achieved, and thereby also that the simulative data basis is sufficient to predict the deformation behavior, respectively, the bending angle of a FRP with the described and investigated material set-up.

#### 3.3.2. Validation of Simulative Investigation via Physical Tests

Because of the aforementioned conservative boundary conditions set for the actuation and failure mechanism in the simulations, the resulting maximal bending angles of the physical tests are higher with less hinge width (Table 4). Calculating similar to the simulation would result in two hinges with a width of 5 cm. Considering the hinge width of 8 cm of the simulation results, the mean value is: 6.5 cm hinge width.

**Figure 15:**
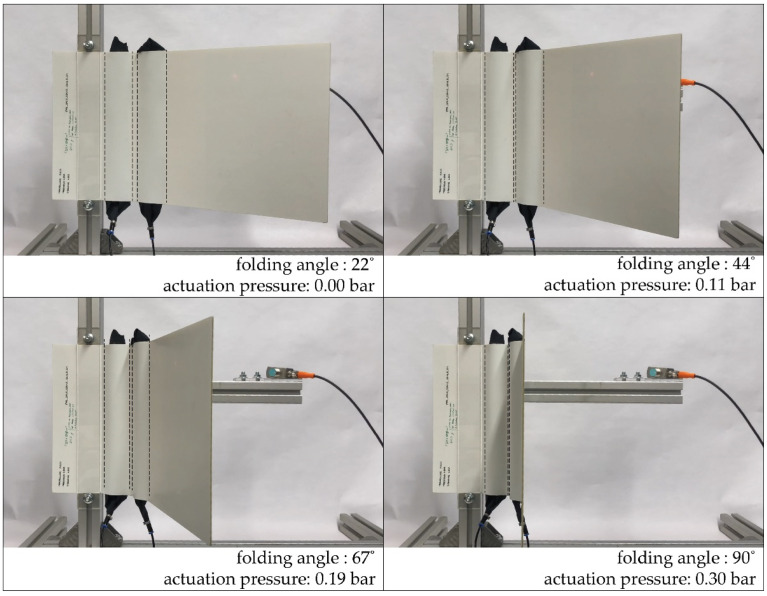
Mechanical testing of the hinge component with two hinges (hinge width 6.5 cm, distance between the hinges 1 cm).

Finally, for the validation of the results derived from the simulation, a test specimen with two side-by-side hinges, each with a width of 6.5 cm and a distance between them of 1 cm, was manufactured and tested in the test rig (cf. Figure 15).

**Figure 16:**
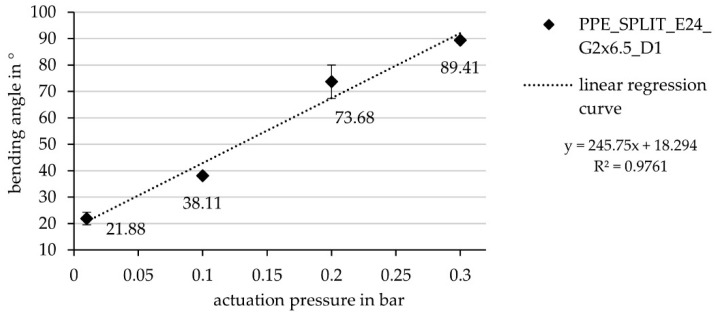
Bending angle as a function of the actuation pressure for the hinge component with two cushions (width per hinge 6.5 cm; distance between hinges 1 cm).

The maximum deformation angle is 90.68° ± 0.47° at a pneumatic pressure of 0.33 bar ± 0.01 bar (Figure 16). The statistical calculation of mean value and standard deviation is based on the first 38 cycles of testing after 150 load cycles, the component delaminated in the bridging area between the two cushion chambers; however, not between the two HHZ99 layers, but between HHZ99 and ET222 (stiff component side).

Thus, the simulation can be confirmed by the tests. It is possible to adjust the deformation angle via the geometric framework conditions of the component. However, the fatigue strength must still be improved for the technical application.

## 4. Discussion

Both the simulation and the physical experiments demonstrate the adjustability of the mechanical properties by adapting the geometry on the basis of the derived regression functions. However, there is a need for further development with regard to fatigue strength, since material failure due to interlaminar adhesion already occurs after 150 cycles. The interlaminar adhesion of this material structure could be improved, for example, by sewing the individual layers together or by Z-pinning. Z-pinning hereby means to introduce small metal or FRP pins through the thickness of the textile stack [16,17]. In contrast to sewing, this can also be done with pre-impregnated fabrics like ET222. A non-material-homogeneous connection between the layers has already been successfully applied in the ITECH Research Demonstrator 2018-19 in the form of screw connections next to the hinges [9]. In contrast to sewing, this can also be done with pre-impregnated fabrics like ET222. However, it would be better to adjust the interlaminar strength via the material properties so that sufficient interlaminar adhesion is present. Conceivably, such strength is achievable by using multilayered woven fabrics, since here the layers are already connected in the Z-direction. 3D woven structures would accordingly offer a possibility to produce components with higher fatigue strength without additional process steps. Additionally, another possibility to avoid the problem of delamination could be an actuation that is distributed over a larger surface compared to the current rod-shaped one. The better pressure distribution due to the larger surface could reduce the load on individual parts. Here, the dynamics of interlaminar adhesion and the geometry of the actuation would have to be further investigated.

The empirical simulations and studies can be used as a data basis for designing new adaptive components and as a benchmark for further investigations. The functionality is generally proven, but the bending angle should be increased for use in various application scenarios. The authors see this research on the bending behavior of a simple geometry as an important step towards the development of adaptive bending-active façade systems. These systems have the potential to be a feasible alternative to conventional mechanical shading systems for complex building geometries. This research contributes to the development of an ongoing project of an adaptive façade system to be installed at the University of Freiburg. This adaptive, bioinspired shading device will consist of FRP elements with asymmetric laminate set-ups and variable stiffness, thereby creating linear hinge zones which will be activated by integrated pneumatic actuators. The façade is being developed by the University of Stuttgart and is intended to be completed by 2022.

## Figures and Tables

**Table 1:** Material properties [12].

ID ^1^	Material	Supplier	Trade Name	Fiber Orientation in °	Tensile Strength in MPa	Young’s Modulus in MPa	Poisson’s Ratio-
ET222	GFRP ^2^	Toray Group Composite Materials (Italy) s.r.l.	ET222	0/90	546.50	16,628.61	0.20
HHZ99	Elastomer	Kraiburg GmbH & Co. KG	Kraibon HHZ9578/99	-	1.89	15.98	0.30
TPU	TPU ^3^	Gerlinger Industries GmbH	4980	-	7.16	126.43	0.35

^1^ short name within the paper, ^2^ glass fiber-reinforced plastic, ^3^ thermoplastic polyurethane.

**Table 2:** Material test overview.

ID ^1^	Hinge Width in cm	Continuous Layers ET222 (±45)°		Width HHZ99 in cm	Layers ET222 (0/90)° (Replacing HHZ99) ^3^
		Laminate (A) ^2^	Laminate (B) ^2^		
PPE_2 + 4	10	2	4	continuous	-
PPE_4 + 8	10	4	8	continuous	-
PPE_6 + 12	10	6	12	continuous	-
PPE_2 + 4_G5	5	2	4	continuous	-
PPE_2 + 4_G7.5	7.5	2	4	continuous	-
PPE_2 + 4_G10	10	2	4	continuous	-
PPE_2 + 4_G12.5	12.5	2	4	continuous	-
PPE_2 + 4_G15	15	2	4	continuous	-
PPE_2 + 4_E15 + 5	10	2	4	15 (+5)	4 layers ET222
PPE_2 + 4_E17.5 + 5	10	2	4	17.5 (+5)	4 layers ET222
PPE_2 + 4_E20 + 5	10	2	4	20 (+5)	4 layers ET222

^1^ short name within the paper; ^2^ cf. Figure 3 (lower stiffness in laminate (A) and higher stiffness in laminate (B)); ^3^ cf. Figure 11 (stiffening of the flaps by substituting HHZ99 with four layers of ET222 with a fiber orientation of (0/90)° in relation to the bending axis).

**Table 3:** Bending angle dependent of hinge widths (simulation) and prediction of bending angle/hinge width (regression formula from Figure 13 used).

Simulations		Prediction Based on Diagram			
Hinge Width in cm	Angle in °	Hinge Width in cm	Angle in °	Angle in °	Hinge Width in cm
5	15.3	6	26.3	35	6.92
7.5	40.7	7	35.7	40	7.50
10	57.0	**8**	**44.0**	**45**	**8.14**
12.5	66.4	9	51.1	50	8.84
15	70.2	10	57.1	55	9.63

**Table 4:** Bending angle dependent of hinge widths (physical tests) and prediction of bending angle/hinge width (regression formula from Figure 13 used).

Physical Tests		Prediction Based on Diagram			
Hinge Width in cm	Angle in °	Hinge Width in cm	Angle in °	Angle in °	Hinge Width in cm
5	43.1	4	37.2	35	3.64
10	63.9	4.5	40.2	40	4.46
15	71.1	**5**	**43.1**	**45**	**5.36**
		6	58.3	50	6.34
		**6.5**	**50.7**	**55**	**7.45**
